# The Application of a Random Forest Classifier to ToF-SIMS Imaging Data

**DOI:** 10.1021/jasms.4c00324

**Published:** 2024-10-25

**Authors:** Mariya A. Shamraeva, Theodoros Visvikis, Stefanos Zoidis, Ian G. M. Anthony, Sebastiaan Van Nuffel

**Affiliations:** † Maastricht MultiModal Molecular Imaging Institute (M4i), Maastricht University, Universiteitssingel 50, 6229 ER Maastricht, The Netherlands; ‡ Faculty of Science and Engineering, Maastricht University, Paul-Henri Spaaklaan 1, Maastricht 6229EN, The Netherlands

**Keywords:** ToF-SIMS, Random Forest, Machine Learning

## Abstract

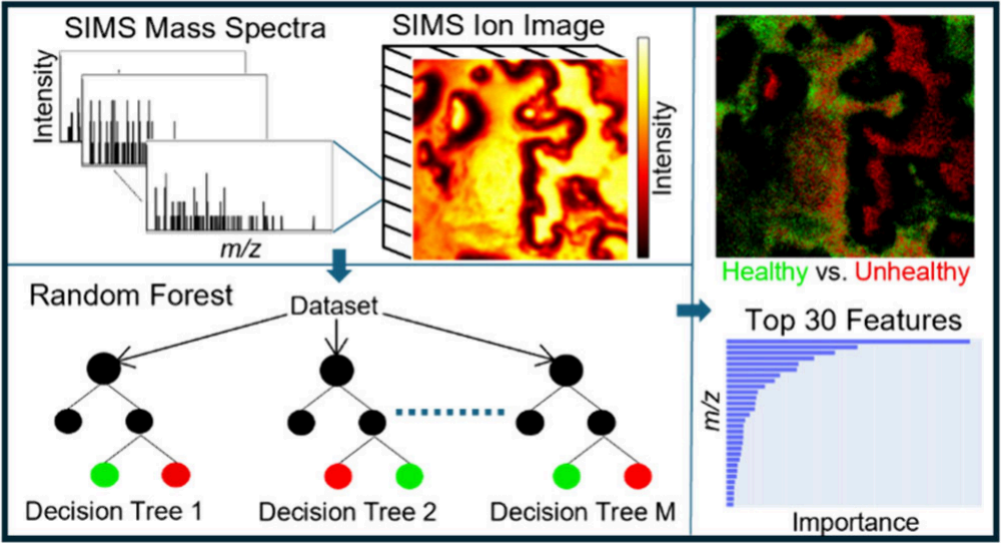

Time-of-flight secondary ion mass spectrometry (ToF-SIMS) imaging is a potent analytical tool that provides spatially resolved chemical information on surfaces at the microscale. However, the hyperspectral nature of ToF-SIMS datasets can be challenging to analyze and interpret. Both supervised and unsupervised machine learning (ML) approaches are increasingly useful to help analyze ToF-SIMS data. Random Forest (RF) has emerged as a robust and powerful algorithm for processing mass spectrometry data. This machine learning approach offers several advantages, including accommodating nonlinear relationships, robustness to outliers in the data, managing the high-dimensional feature space, and mitigating the risk of overfitting. The application of RF to ToF-SIMS imaging facilitates the classification of complex chemical compositions and the identification of features contributing to these classifications. This tutorial aims to assist nonexperts in either machine learning or ToF-SIMS to apply Random Forest to complex ToF-SIMS datasets.

## 1 Introduction

Time-of-flight secondary ion mass spectrometry (ToF-SIMS) has been used extensively for several decades for the surface analysis of a wide range of inorganic and organic material systems due to its chemical specificity and sensitivity.^1−6^ Mass spectrometry images are generated by raster scanning a sample in two dimensions using a focused primary ion beam and acquiring mass spectra for each pixel.^7^ Additionally, it is possible to include a third dimension of depth by removing material by sputtering and performing scans at different depths within the sample, thereby creating a 3D image stack. The ToF-SIMS spectra of most organic materials are complex because primary ion beams often cause significant fragmentation.^8^ In numerous cases, molecular ions have low intensities or are not observed at all in the ToF-SIMS spectra. Thus, much of the information about the surface chemistry is obtained via fragment ions, which complicates the data interpretation. The superposition of various fragmentation patterns, especially when analytes share similar structural features, leads to convoluted datasets. Furthermore, secondary ions yields do not always correspond to their abundance in the sample.^9^ As a result, ToF-SIMS is nonquantitative, especially in the case of molecular imaging below the static limit.^8,9^ Nonetheless, ToF-SIMS imaging has become a potent tool that enables untargeted analysis of molecular species, for example in the contexts of material sciences and biomedical research.^10−12^

The analysis of ToF-SIMS images poses several challenges. First, electric field effects introduced by surface topography and charging can introduce mass shifts and signal loss that may further complicate data analysis.^13−17^ These field effects can be reduced via instrument designs with a pulsed analyzer or via the use of an extraction delay in instruments with pulsed primary ions.^18^ Surface charging can also be compensated using dedicated charge neutralization hardware.^19,20^ Second, the nature of the noise should also be considered; there is a consensus that data should be scaled in a manner that is consistent with its noise.^21−24^ When techniques rely on ion counting as is the case for some time-of-flight mass analyzers,^22−24^ the noise follows a Poisson distribution, making it proportional to the square root of the average number of counts and, with enough counts, should approach normality.^24^ Unfortunately, ToF-SIMS data, especially individual pixels in ToF-SIMS images, frequently deviates from normality because of low ion counts.^25,26^ Third, nonlinearity can be introduced by detector saturation and matrix effects. Care should be taken to avoid detector saturation during data collection. Counting statistics of a Poisson process with dead time refers to the statistical treatment of event counting where the events follow a Poisson distribution but are subject to a dead time during which the detection system is unable to register subsequent events. Dead time corrections (using Poisson statistics) can also be used to reduce such nonlinearity issues. This correction is essential for maintaining the accuracy and reliability of quantitative measurements in systems where event rates can be high, such as in mass spectrometry imaging.^23^ Matrix effects are, however, still poorly understood and can be difficult to avoid.^27^

Conventionally, ToF-SIMS imaging requires user interpretation, which benefits from *a priori* knowledge of the sample. As user interpretation is often slow and can require extensive training, machine learning (ML) can be used to help analyze ToF-SIMS data.^28^ These approaches are increasing in use as mass spectrometry imaging (MSI) datasets, and ToF-SIMS MSI data in particular, are highly multidimensional, typically with 10^4^–10^6^ bins per pixel, which can lead to individual images to be many GBs or even TBs.^29^ MSI datasets, ToF-SIMS datasets in particular, and ML are a good combination as these are large sample procedures.

Unsupervised ML methods have several potential advantages in exploratory research, namely, visualization, dimensionality reduction, image segmentation, unmixing, pattern extraction, and denoising.^29^ Unsupervised ML methods can be divided into several specific subbranches: factorization methods, partitioning and clustering methods, and manifold learning methods. Examples of factorization methods are principal component analysis (PCA),^30−32^ weighted PCA (w-PCA),^23,33^ independent component analysis (ICA),^34^ maximum autocorrelation factor (MAF),^25,35,36^ non-negative matrix factorization (NMF),^37−41^ multivariate curve resolution (MCR)^42,43^ and multivariate curve resolution-alternating least-squares (MCR-ALS),^44,45^ probabilistic latent semantic analysis (pLSA),^41,46^ CX/CUR matrix decomposition,^47^ dictionary learning or molecular dictionary learning (MOLDL)^48^ as well as others.^49,50^ PCA is among the most used multivariate analysis techniques for SIMS-based MSI. Using PCA, high-dimensional data can be decomposed into a lower-dimensional space.^26^ PCA can be effective for the efficient retrieval of sources of variation in the data, and the investigation of the linear correlations and trends between mass peaks within an MSI dataset.^31,51,52^ However, PCA provides limited spectral information compared to other matrix decomposition methods and can be difficult to interpret.^29^ Partitioning & clustering methods are a second widely used class of algorithms for exploratory MSI analysis such as k-means,^32,53−55^ hierarchical clustering (HC),^29,56,57^ bisecting k-means,^58^ high dimensional data clustering (HDDC)^59,60^ and soft segmentation techniques such as fuzzy c-means clustering (FCM),^61^ AMASS,^62^ latent Dirichlet allocation,^63^ and spatial shrunken centroids.^64^ The linear nature of matrix factorization methods makes them less appropriate for nonlinear data. Nonlinear data can be analyzed by manifold learning methods. Manifold learning methods include t-distributed stochastic neighbor embedding (tSNE),^65^ uniform manifold approximation and projection (UMAP),^66^ self-organizing maps (SOMs), autoencoder (AE),^67^ and Kohonen (neural) networks.^68^ It has been demonstrated that AE classifies MALDI imaging data from biological samples more effectively than PCA and NMF. Additionally, Matsuda showed that AE segmented ToF-SIMS data of human skin with greater detail than PCA or MCR.^69^ Furthermore, Aoyagi et al. reported that AE offered more accurate results for quantitatively analyzing SIMS data from organic mixtures with matrix effects.^70^

Supervised ML methods are widely used to identify molecular signatures and disease diagnosis^71,72^ and include partial least-squares (PLS) regression,^73,74^ Random Forest (RF),^46,74,75^ Markov random field (MRF),^74^ logistic regression (LR),^71^ gradient boosting,^76^ support vector machine (SVM),^77^ and others.^76,78^ Supervised machine learning models the relationship between input data and output data and requires human annotation in comparison to unsupervised ML where no human annotation is needed. Another subset of ML is deep learning and neural network methods with artificial neural networks (ANN).^79−82^ For instance, an ANN-based supervised learning method is useful for both quantitative and qualitative analysis of SIMS data from organic mixture samples affected by matrix effects.^83^ All the above-mentioned ML methods have been discussed in reviews written by Mehta et al.,^21^ Graham and Castner,^26^ Verbeeck et al.,^29^ Jetybayeva et al.,^78^ and Gardner et al.^81^

Even though the RF algorithm has a relatively low feature and sample robustness, it has many advantages such as medium robustness to overfitting, outliers, and mislabeled data. An example of the application of RF is an extensive interlaboratory study, collecting over 1,000 spectra from six peptide models using instruments from 27 different ToF-SIMS setups across 25 institutes worldwide. The RF algorithm was trained with 20 amino acid labels to classify and identify the peptides from the spectral data. The method demonstrated its effectiveness in determining the amino acid composition of unknown peptides and its potential to uncover novel chemical features within ToF-SIMS.^75^

In summary, RF provides high classification accuracy and information about feature importance. Furthermore, it can handle the nonlinearity introduced by for example matrix effects.^84,85^ This tutorial seeks to aid nonexperts in either RF or ToF-SIMS. We summarize decision trees and Random Forest theory and provide guidelines on their application and use for ToF-SIMS imaging data. We also present a practical example of the application of RF to a ToF-SIMS dataset.

## 2 Theoretical Background

### 2.1 Decision Trees

**Figure 1:**
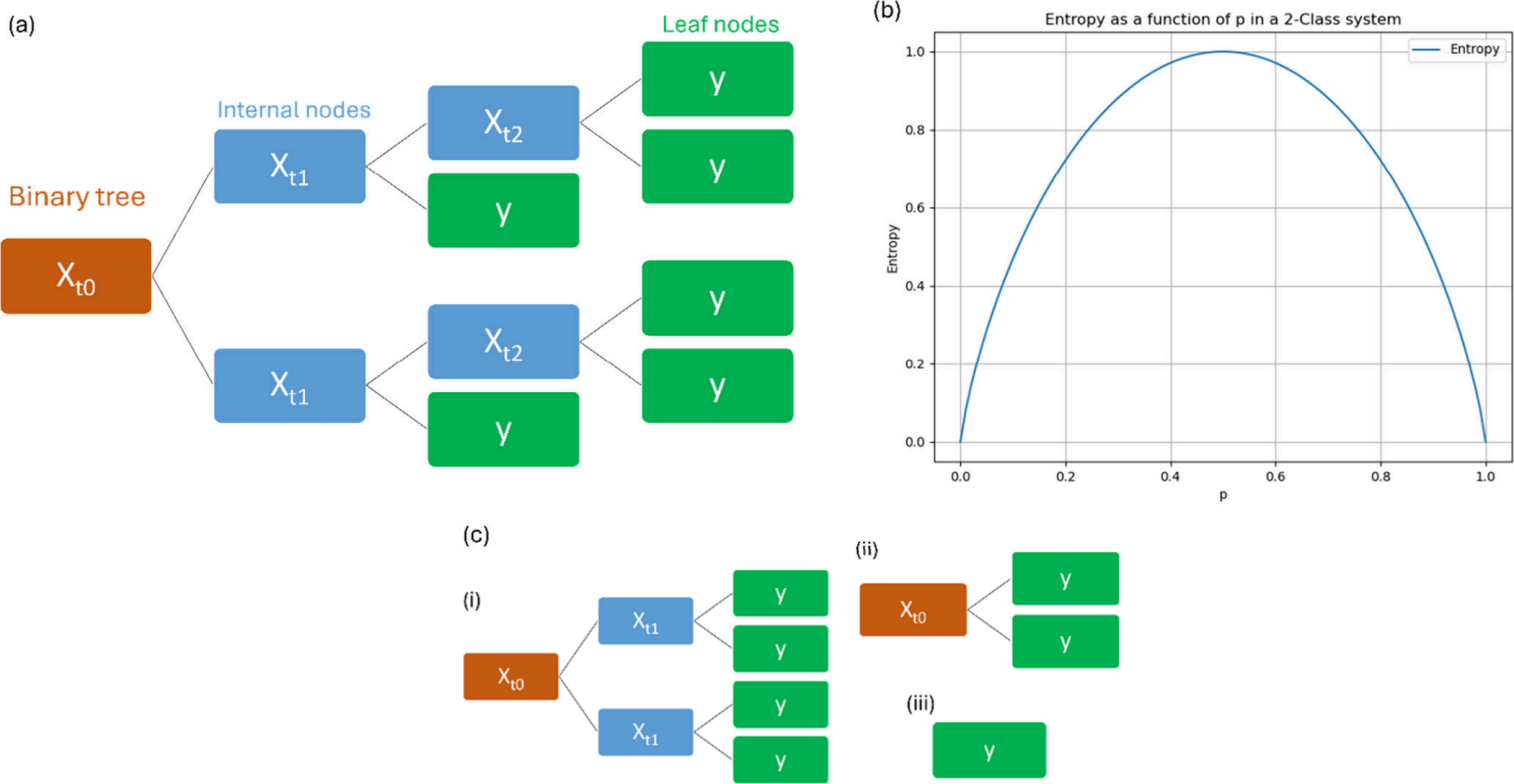
Schematic overview of decision trees. (a) The binary tree (*X*_*t*_0__) splits into two internal nodes, with the node *X*_*t*_1__ being the parent of *X*_*t*_2__. This process continues, with each node branching further, until a terminal node without children (leaf node) is reached. (b) The entropy function as an impurity measure: (1) highest when the distribution of node proportions is uniform (*p*_*i*_ values are equal), (2) lowest when the probability of a specific class *p*_*i*_ is 1, and (3) symmetric under permutation of *p*_*i*_ values. (c) Pruning tackles overfitting by reducing the size of decision trees, recombining a large tree upward (i, ii, iii), thus decreasing the sequence of subtrees. By eliminating unnecessary branches and nodes, classification accuracy can improve.

Decision trees were first published by Morgan and Sonquist in 1963,^86^ who published an automatic interaction detector (AID) tree-based technique for managing multivariate nonadditive effects in survey data. This publication was followed by several more advancements.^87,88^ Throughout the 1970s, Breiman,^89^ Friedman,^90^ and Quinlan^91^ independently proposed similar algorithms for the induction of tree-based models. A decision tree is a type of supervised learning algorithm with advantages such as handling heterogeneous data, robustness to outliers and to noise due to feature selection, and applicability both for classification and regression tasks.^92−96^*Classification and Regression Trees (CART)*, *Iterative Dichotomiser 3 (ID3)*,^97^ and *C4*(98) are examples of decision tree algorithms. In this tutorial, we focus on *CART* with special attention to classification trees.^92^ The idea behind the decision-tree model involves approximating the Bayes model partition by recursively splitting input space *X* into subspaces and then assigning constant prediction values. Let us define a rooted tree as a graph *G* = (*V*, *E*), where the set of edges (*E*) is directed away from the root (Figure 1). Any two vertices (*V*) (or nodes) in a graph have only one path connecting them. A tree’s root node splits into two or more sets (or subpopulations) with increased homogeneity if there exists an edge from *t*_1_ to *t*_2_ (i.e., (*t*_1_,*t*_2_) ∈ *E*), then node *t*_1_ is the parent of node *t*_2_ while node *t*_2_ is the child of node t_1_. These new subpopulations or internal nodes, which have one or more children, continue splitting or branching until a homogeneous terminal node with no children, also referred to as a leaf node, is reached. The objective is to train a decision tree to generate rules to predict the target variable.

In general terms, an impurity measure *i*(*t*), using the framework of Breiman in 1984,^92^ can be defined as a function that assesses the goodness of any node *t*.

The most common impurity criteria used for classification trees are the Gini impurity based on the Gini index^99^ and the Shannon entropy.^100^ However, there are other metrics like cross-entropy,^101^ and mutual information^102^ that are also crucial. Impurity criteria are used to measure how well a split separates the classes in a dataset. The Gini impurity measures how often a randomly selected element of a set would be mislabeled if it were labeled randomly and independently based on the label distribution within the set. It is calculated by summing the pairwise products of the probability of choosing an item of a particular class and the probability of miscategorizing that item for each class label. Shannon entropy is another such measure, quantifying the uncertainty or impurity in the data. The Shannon-entropy-based criteria measure the uncertainty of the target variable within a given node. As the impurity-based on entropy decreases, the information gained about the target variable by splitting the node increases, which is commonly known as information gain.^103,104^ Cross-entropy measures the difference between two probability distributions: the true distribution of the data and the predictions made by the model. In the context of decision trees, cross-entropy can be used to evaluate how well the tree’s splits are classifying the data. Lower cross-entropy indicates a better-performing split. Mutual Information quantifies the amount of information gained about one variable through another variable. In decision trees, it helps determine which feature to split on by measuring how much knowing the value of a feature reduces the uncertainty of the target variable.

The Classification and Regression Trees (CART) algorithm uses Gini impurities to decide where to split the data. However, other algorithms apply different methods to evaluate the purity of a node. These methods include Oblique Classifier 1 (OC1),^105^ Chi-square automatic interaction detection (CHAID),^93,106^ multivariate adaptive regression splines (MARS),^107^ and Conditional Inference Tree.^108^ Each of these functions uses its impurity criteria that exhibit the necessary properties to be an impurity function to determine the best way to split the data in a decision tree.^109^

In decision tree classification, the likelihood of incorrect predictions can be measured by calculating the misclassification rate. As more splits are added to a tree, this rate typically decreases, suggesting that the tree becomes more accurate, which might lead to overfitting. Conversely, if we incorporate too few terminal nodes, it may lead to underfitting. Finding the ideal trade-off between a tree that is neither too deep nor too shallow is therefore essential.

Both overfitting and underfitting result in errors that hinder supervised learning algorithms from generalizing beyond their training set. Overfitting leads to high variance, which is an error caused by the algorithm’s sensitivity to small fluctuations in the training set. High variance can occur when the algorithm models the random noise present in the training data. Underfitting leads to high bias, which causes errors from incorrect assumptions made by the learning algorithm. High bias can cause the algorithm to overlook the relevant relationships between features and the target outputs. This is commonly known as the bias-variance trade-off.^110^ User-defined “hyperparameters” (parameters that specify details of the learning process) can be tuned to find the right trade-off.

Pruning can be used to tackle the issue of overfitting in decision trees. This technique involves reducing the size of decision trees by recombining a large tree upward, thereby decreasing the sequence of subtrees.^111^ The objective of pruning is to determine the optimal model complexity that minimizes both error sources simultaneously. There are two techniques for pruning a decision tree, namely, postpruning (backward pruning) and prepruning.^112−114^ However, as we will see in the next section, pre- or postpruning is no longer necessary to achieve good generalization performance in the context of an ensemble of decision trees. The CART system^92^ employs a tree pruning method that is based on trading off predictive accuracy versus tree complexity; this trade-off is governed by a parameter that is optimized using cross-validation (CV) (Figure S1), which can partition the dataset in different ways.^115,116^

CV schemes are exploited to increase the variation in the training and testing data and to reduce the influence of the data split on the output testing statistics.^117^ There are two main cross-validation methods, namely, nonexhaustive CV and exhaustive CV.^118,119^ Nested CV has become a popular method for performing external CVs and improving the estimation of unbiased performance.^116,120^ An example of an exhaustive CV is a leave-one-out CV (LOOCV) (Figure S1).^120^ Since there is a good chance of finding similarities between the training set and the testing sets, LOOCV is known to generate overoptimistic estimates.^115^

### 2.2 From Trees to Random Forest

An ensemble of decision trees is referred to as a Random Forest.^121−125^ The main differences between ensemble methods and Random Forest methods are based on how baseline models are trained and combined. The most widely used ensemble methods are bagging, stacking, and boosting. There are many reviews in the literature about ensemble techniques.^127−130^ However, in this tutorial, we will focus on specific ensemble method described by Breiman in 2001, which is referred to as Random Forests.^131^

**Figure 2:**
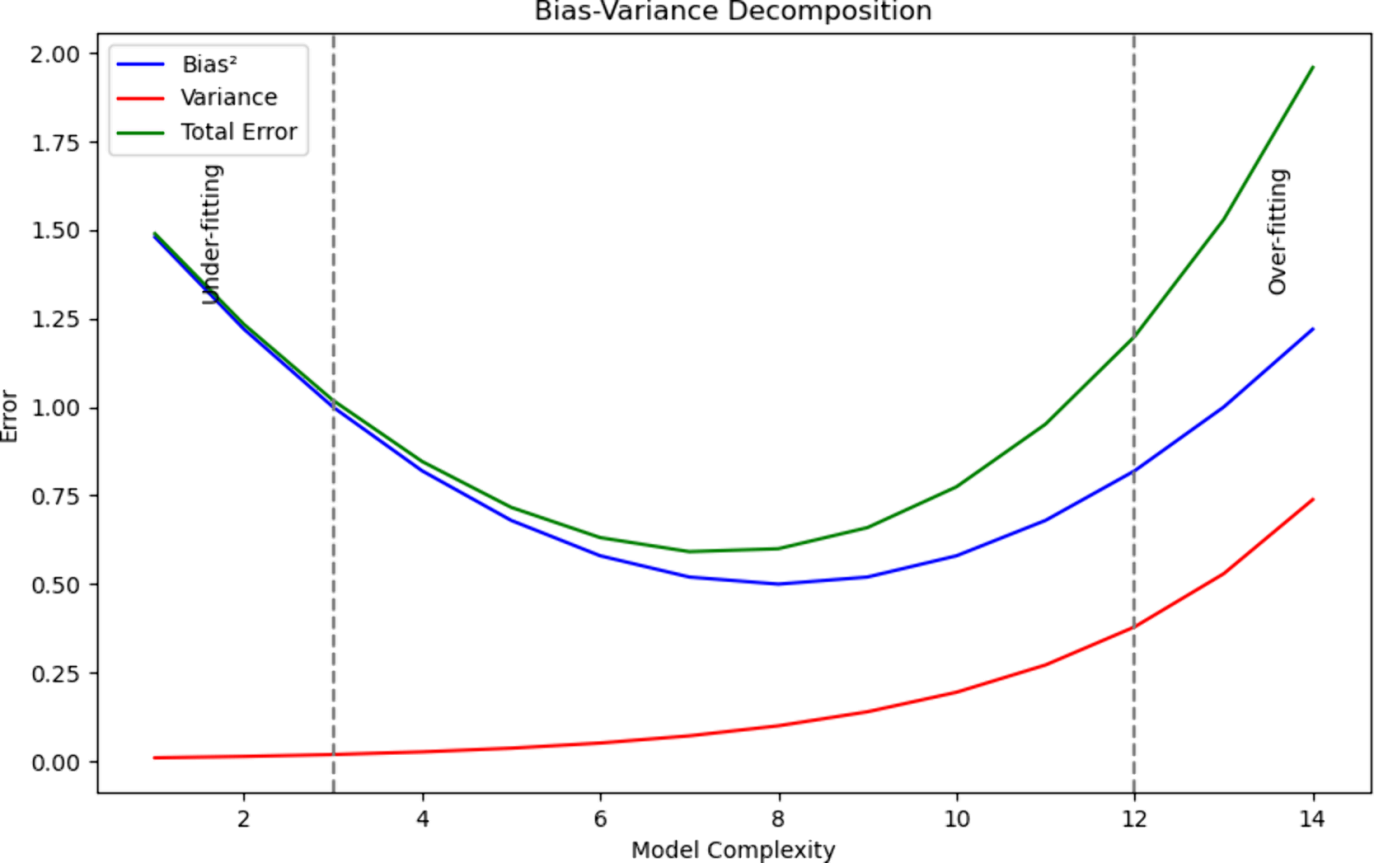
Bias-variance decomposition: This figure illustrates the bias-variance decomposition as a function of the model complexity. The blue line represents the squared bias, which is high for simple models (underfitting) and decreases as the model complexity increases. The red line represents the variance, which is low for simple models but increases with model complexity, indicating overfitting. The green line represents the total error, which is the sum of the bias squared and variance. Vertical dashed lines mark the regions of under- and overfitting. Underfitting occurs at low model complexity with high bias and low variance, while overfitting occurs at high model complexity with low bias and high variance. The optimal model complexity balances bias and variance, resulting in the lowest total error.

The bias-variance decomposition for the squared error loss of the generalization error was first proposed by Genman^132^ in the context of neural networks. Similar decompositions for the expected generalization error based on the zero-one loss have been proposed in the literature, drawing a direct analogy with the bias-variance decomposition for the squared error loss by redefining the concepts of bias and variance in the case of classification.^122,126,133−136^ The bias-variance decomposition is a useful tool for diagnosing underfitting and overfitting (Figure 2).

Reducing the prediction variance by implementing ensemble methods, Random Forest is a reasonable strategy for decreasing generalization error, assuming that the corresponding bias can be maintained at the same level or not increased excessively. Breiman showed in 1996 that the average model has a lower expected generalization error.^126^ Intrinsically, bagging is classified by averaging combined models built from a bootstrap sample *L*^*m*^ for *m* = 1, ..., *M* of the training set. *L*^*m*^ builds replicates of *L*, given a training dataset with *N* observations and a binary target variable (*x*, *y*), drawn at random but *with replacement* from *L*.^137^ When numerous bootstrap samples are generated from a large sample size after *N* draws with replacement, the probability of never having been selected is ; therefore, each bootstrap sample contains  of the original observations.^137,138^ Because bagging produces unique models that vary from one bootstrap sample to another, they are more likely to benefit from the process of averaging. Therefore, this approach enhances model performance by reducing the variance without increasing bias. Breiman demonstrated that classification accuracy and stability were significantly enhanced when averaging classification outcomes obtained from multiple bootstrap samples of the original training set. However, if the learning set *L* is small, subsampling 67% of the objects may result in an increase in bias due to a decrease in model complexity. This bias may be too large to compensate for the decrease in variance, leading to a worse performance overall. Despite this flaw, bagging is an effective method. Subsequently, the use of bagging was expanded to any kind of model–i.e. not only decision trees, and to generalized statistical analysis, which showed sampling with and without replacement yielded equivalent improvements.

In 2001, Breiman^131^ combined bagging with feature bagging,^123^ which resulted in a method known as Random Forests. Feature bagging is an altered tree learning algorithm that chooses a random subset of features at each potential split. Consequently, each tree is generated from a bootstrap sample of training data and uses a random sample of features for each split. This approach effectively reduces both variance and bias. The improved reduction in variance is attributed to the increased independence of the trees, which is achieved through a combination of bootstrap samples and a random selection of features. Similarly, the bias is decreased because there are often a few dominant features that consistently outperform their counterparts during the decision tree fitting process. By employing feature bagging, a vast number of predictors can be considered, allowing local feature predictors to contribute to the construction of the tree. The construction of a classification tree is like the construction of Breiman’s original decision tree.^131^ It begins with the root that contains all of the training samples. A subset of features is chosen randomly at each node, and the subset’s characteristic that permits the greatest class separation in the sample set at that node is identified. Next, the node is split into two child nodes. The process is then repeated, until the tree is fully grown; that is, all leaf nodes contain samples from one class only. The trees grown are not pruned.^131^ This entire process is repeated many times. After many trees are generated, each observation is assigned a final class by a plurality vote.^131^ Breiman empirically demonstrates that Random Forest outperform boosting^125^ and arcing algorithms,^123^ both of which are geared toward reducing bias, whereas forests concentrate on reducing variance.

As we sample with replacement, there will be  observations that are not part of the bootstrap sample, known as out-of-bag (OOB) observations. These observations can be considered as a test dataset and dropped down the tree, enabling us to estimate the prediction error. The out-of-bag error refers to the average prediction error on each training sample using only the trees that did not include that specific training sample in their bootstrap sample. The proximity measure^139^ between two sample points is another useful feature of tree-based ensemble methods.

The importance of the different features for the classification can be estimated with the permutation accuracy criterion.^131^ However, little is known about the variable importance calculated by Random Forest, and to the best of our knowledge, Ishwaran’s work in 2007 is the most fundamental work devoted to the theoretical analysis of tree-based variable importance measures.^140^ The commonly used methods to estimate the prediction accuracy or variable importance include permutation importance,^131^ Impurity importance^141^ if the Gini index is being used as an impurity function, Actual impurity reduction importance,^142^ and others.^141^

It is worth mentioning that Random Forest, which is a bagging extension of ensemble methods, is one but not the only example of the application of the ensembles of Decision Trees. There are also Extremely Randomized Trees. Similarly to Random Forest, a random subset of possible features is used, but instead of finding the most optimal thresholds, threshold values are randomly selected for each possible feature, and the best of these randomly generated thresholds is chosen as the best rule for splitting a node. This usually reduces the model variance slightly at the expense of a slightly larger increase in the bias. Boosting is another ensemble strategy for generating a set of predictors, but the accuracy of RF is comparable to that of boosting.^143^

There are also several enhancements to the boosting based on different boosting algorithms such as AdaBoost, Gradient Boosting, XGB, Light GBM, and CatBoost. Stacking or stack generalization is also an ensemble technique. While specific details regarding the algorithms are out of the scope of this review, the application of Random Forest is discussed in the following section.

### 2.3 Application of Random Forest to ToF-SIMS Data

Random Forest has been applied to mass spectrometry imaging datasets obtained by ToF-SIMS^71,75^ and other MS methods such as MALDI^46,144,145^ or some others.^78,146,147^

As mentioned in the Introduction, acquiring good quality data using an appropriate experimental setup can already avoid issues such as field effects introduced by topography and surface charging and nonlinearities caused by detector saturation. Crucial preprocessing steps can include but are not limited to mass calibration, normalization, baseline correction, denoising, peak picking, and dimensionality reduction. Considering the nature of ToF-SIMS data is necessary while doing all the preprocessing steps. Incorrect mass calibration would not affect the Random Forest algorithm directly, but a correct calibration of the mass spectra is, of course, important for the interpretation of the output by the analyst. Green et al. provide a useful guide for ToF-SIMS mass calibration.^148^ Regarding mass calibration, peak picking, and mass bin width, Madiona et al.^149^ evaluated bin size using Shannon entropy, while Lang et al.^76^ proposed a conversion method for mass spectra. Normalization by the total ion count (TIC) per pixel effectively mitigates variations in the secondary ion signal due to differences in topography, sample charging, or instrumental conditions such as variations in primary ion current or detector efficiency, facilitating comparison across measurements taken on different days. Nevertheless, caution is advised when applying TIC normalization, because it may produce misleading results. Baseline correction is usually unnecessary in ToF-SIMS, unlike other MS techniques due to the low levels of chemical noise.^150^ Denoising also might be considered as one of the preprocessing steps, because the performance of ML models is driven by the bias-variance trade-off. In particular, Haar wavelet denoising, or the down-binning of specific *m*/*z* images, is a commonly used technique for ToF-SIMS imaging.^151,152^ While performing peak picking it is also important to take into account the large disparity of signal-to-noise ratio (SNR), and noise, which is still a problem when it comes to automated peak identification in SIMS.^150^ The use of derivative spectrometry based on the continuous wavelet transform (CWT) usually facilitates peak detection in ToF-SIMS.^151^ Collinear features such as *m*/*z* channels of a single peak or fragments of the same molecule could potentially split the importance among themselves, reducing the clarity of results. If a SIMS imaging dataset is acquired on a system with a detector that follows Poisson statistics, then before any dimensionality reduction, a valid preprocessing step should include Poisson scaling,^23^ because principal component analysis (PCA)^30−32^ works optimally on data with a Gaussian distribution and weighted PCA (w-PCA)^23,33^ alleviates the effects of the Poisson distributed noise. However, it is important to consider one potential drawback of scaling, namely its tendency to reduce sparsity in MSI datasets, preventing the use of efficient sparse representations of the datasets which would significantly reduce computational demands.^153^

For MSI datasets, the mass channels of a mass spectrum can be considered the features, and the individual pixels are considered separate samples. Class labels need to be assigned to each pixel in a training dataset. One of the important considerations is the correct selection of data-partitioning schemes. The output from the RF model may serve as an input for subsequent analyses, and a bad data-partitioning scheme can adversely affect these downstream processes, leading to potential misinterpretation. As a rule of thumb, a training set, which should be independent of the testing set, is usually taken as 70% of the samples and testing as the remaining 30%.^154^ However, some data-partitioning schemes are only effective if the size of training and testing sets are big enough and representative of the parameter space, namely, the single train–test split.^115,116^ When a dataset is small, the k-fold cross-validation estimate is usually preferred over the test sample estimate. Moreover, another important consideration concerning the application of RF to ToF-SIMS data is feature extraction and selection. One strategy is to use the entire mass spectrum either by using the individual mass channels or by down-binning them to reduce their number. In this case, noise is included in the feature space. The alternative strategy is to perform a peak search, enabling the creation of a better-performing model, which might introduce some user bias if done incorrectly. Although RF classifiers exhibit notable robustness to overfitting, outliers, and mislabeling, enough extreme outliers and mislabeled samples in the training dataset may still impact the performance of the RF model. Moreover, they can lead to overfitting or certain (sub)trees within the RF to focus on features that are not representative of the classification problem. Outliers can compel trees within the Random Forest to grow deeper to isolate these points, thus increasing the computational complexity. The removal of outliers promotes more balanced trees, streamlining the model training process and reducing computational demands. Moreover, initially during training, the optimal hyperparameters of RF should be determined. The hyperparameters that should be considered when training a model: n_estimators, the number of trees in the forest; criterion (“gini”, “log_loss” or “entropy”), max_features, the number of features to consider when looking for the best split (for classification tasks, the default value is √*n* where *n* is the number of features); min_samples_leaf, the minimum number of samples required to be at a leaf node; max_depth, the maximum depth of the tree. As an example, various numbers of trees (in the range of 10–1000) have been reported for robust RF.^155−157^ The latter can be tuned, for instance, through consecutive repetitions, calculating some quality metrics such as mean value and standard deviation of the out-of-bag error,^158^ or through a CV.^159^ Other hyper-parameters such as the number of variables used in each node can be optimized similarly.^156,160,161^ Linear combinations of variables (for example, using dimensionality reduction algorithms) can be also used to reduce the number of features to improve results and computation times at the cost of interpretability.^147,158^ It is sometimes crucial to decrease the number of variables because of the high collinearity of ToF-SIMS data to get a robust and reproducible model.^162^ An optimal number of variables can be determined via a nested cross-validation process.^163^

## 3 Practice Example Using a ToF-SIMS Dataset

In this section, we will provide a simple demonstration of Random Forest applied to ToF-SIMS image data. In a previous publication, Random Forest was successfully used to identify potential marker ions for pulmonary arterial hypertension (PAH) in human lung arteries for the MALDI dataset.^144^ In that study by Van Nuffel et al., a ToF-SIMS imaging dataset of control and PAH-related arteries was collected but had not been analyzed using Random Forest.

### 3.1 Experimental Section

#### 3.1.1 Biological Sample Preparation and ToF-SIMS Analyses

For the sample preparation of the human lung tissue sections and instrumental set up including experimental parameters, we refer the reader to the previous study,^144^ where it has been described in detail. Table S1 provides an overview of the samples used in the example. For this demonstration, 8 negative polarity ToF-SIMS images of 4 control arteries and 4 occluded PAH arteries are selected.^144^

#### 3.1.2 Data Preprocessing

The ToF-SIMS datasets are first internally calibrated with the following ions: CN^–^, CNO^–^, C_14_H_27_O_2_^–^, C_16_H_31_O_2_^–^, and C_18_H_35_O_2_^–^ using SurfaceLab software (IONTOF GmbH, Germany) (Figure S2). Then, the calibrated ion images were converted to ImzML and loaded in Python (3.12.3), where the processing was performed by using custom-made scripts. The script iterates through the files, loading the data matrices and spatial coordinates, while also assigning labels based on the filenames—labeling “Control” data as 0 and “PAH” data as 1. After collecting all data matrices, it combines them into a single sparse matrix (combined_matrix). The data analysis pipeline included peak picking with further extraction of peaks. The peak list includes 608 peaks in the *m*/*z* range of 0–1850 Da. Additionally, it compiles the mass axis, spatial coordinates, and labels and returns them for subsequent analysis steps. This consolidated dataset, along with the corresponding metadata, forms the foundation for later stages of the analysis pipeline.

Finally, the Compressed Sparse Row (CSR) matrix was normalized by scaling its values to fall within the range of 0 to 1. It does this by first identifying the maximum and minimum values within the matrix, which define the data range. Then, each element in the matrix is scaled by subtracting the minimum value and dividing by the difference between the maximum and minimum values. The ion image of palmitic acid at *m*/*z* 255.2 is used as an indicator of biological tissue to remove background pixels from the image data. The signal of palmitic acid is extracted from a data matrix by selecting the *m*/*z* values within a defined tolerance around a target *m*/*z* value (±0.5). This ion image is then thresholded based on a specified intensity threshold (0.05), creating a binary mask that identifies the pixels with ion intensity above the threshold. The thresholded image is then returned, and the indices of the “active pixels” that meet the threshold criteria. The function takes these results and creates visual representations of the ion image and the thresholded image. It reshapes the data into a 512 × 512 pixel grid, corresponding to the spatial coordinates of the sample, and uses the HoloViews library to create two images: one showing the original ion intensities and another highlighting the thresholded regions in a binary format (Figure S3).

#### 3.1.3 Random Forest Implementation

The Random Forest is implemented using the scikit-learn class library via the RandomForestClassifier. It begins by splitting the input dataset (active pixels) and labels (active labels) into training and testing sets. The RF training procedure included 10% active pixels. The function then initializes the RandomForestClassifier with predetermined hyperparameters and fits the model to the training data.

To optimize different hyperparameters, the RandomForestClassifier function uses stratified k-fold cross-validation to evaluate the model’s performance with varying values of a specific hyperparameter, namely the number of trees (n_estimators), maximum depth of trees (max_depth), and the minimum number of samples required to be at a leaf node (min_samples_leaf) are used. For that it iterates over a grid of values for the number of trees and trains the RandomForestClassifier using each value, tracking the model’s accuracy on both training and cross-validation sets. It identifies the optimal number of trees that result in the highest cross-validation accuracy. Similarly, max_depth optimization and min_samples_leaf optimization iterate over different values for the maximum tree depth and the minimum samples per leaf, respectively, each time identifying the best-performing model based on cross-validation accuracy. For each hyperparameter setting, the functions collect and calculate metrics, including training accuracy, cross-validation accuracy, and the standard deviation of the accuracies. These metrics are then used to generate plots, which visually represent the model’s performance across the different hyperparameter values (Figures S4–S9). The plots include training and cross-validation accuracy as well as error bands to show variability. Each function returns the best classifier trained during the process, providing an optimized model configuration for further use.

In order to address multicollinearity, the visualization function is designed to analyze its effects by iteratively removing highly correlated features and observing changes in feature importance and model accuracy. For each threshold value that defines the level of acceptable multicollinearity, the function reduces the feature set by eliminating highly correlated features. It identifies and removes features from the training dataset that exhibit high collinearity based on a chosen arbitrary correlation threshold. Here, 0.30 and 0.90 were used. It starts by computing the correlation matrix of the input feature matrix, which measures the pairwise correlations among all features. Then, it extracts the upper triangle of this matrix, excluding the diagonal, to focus only on the unique feature pairs. Using the specified threshold, the function identifies pairs of features with a correlation coefficient greater than the threshold, indicating strong collinearity. It then determines which features to remove to reduce redundancy in the dataset. The function returns a new feature matrix containing only the remaining, less correlated features along with the indices of the removed features. The reduced datasets are then used to train and evaluate the model multiple times, capturing performance metrics such as accuracy, training time, and feature importance. These metrics are tracked across different thresholds to understand the impact of multicollinearity on the model’s performance. The function then compiles the feature importance scores into a data frame and assigns a consistent color map for visualization. It plots the feature importance to show how the significance of individual features changes with varying thresholds for multicollinearity. Additionally, it tracks the top features across thresholds, highlighting how the importance of these key features evolves as more correlated features are removed. The function plots performance metrics to provide insights into how multicollinearity affects the model’s accuracy and training efficiency (Figure S9).

**Figure 3:**
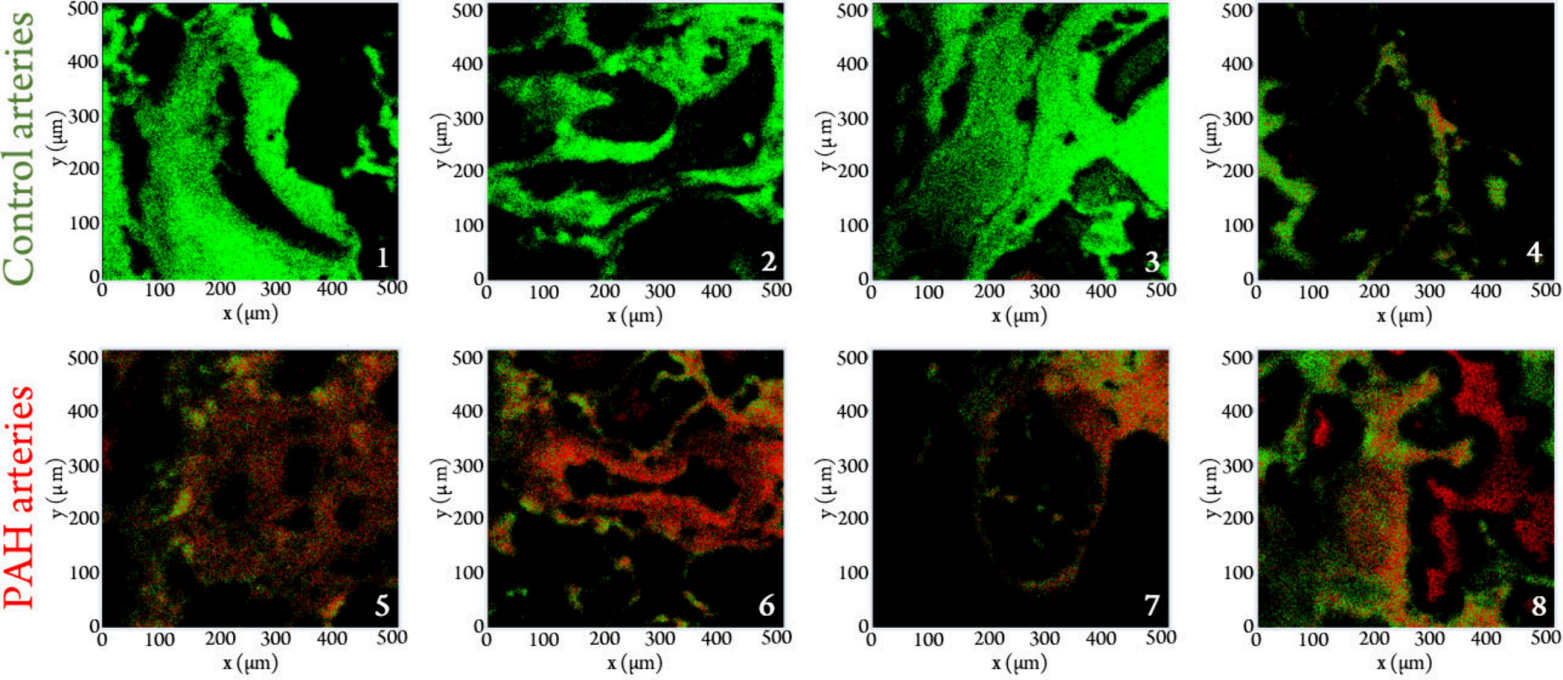
RF classification results of human lung tissue (green pixels classified as normal; red pixels classified as PAH-related). Top row: normal control arteries; bottom row: occluded PAH arteries.

After all the optimizations, the function predicts the labels for the test set and evaluates the model’s performance by calculating the accuracy and generating a detailed classification report. The top important features are visualized by extracting the feature importance from the classifier, which indicates how much each feature contributes to the model’s decision-making process (Figures S10 and S11). For further visualization, the function takes the trained optimized Random Forest classifier and uses it to predict the classes for all active pixels in the dataset. It starts by extracting the relevant pixel data from the combined data matrix and applying the classifier to obtain predicted labels. The function then iterates over each image, using the spatial coordinates to place the classified pixels in their correct positions on a blank image grid. Pixels predicted as “healthy” (label 0) are colored green, while “unhealthy” pixels are colored red (Figure 3).

### 3.2 Results and Discussion

The accuracy of CV ideally should reach an asymptote with a certain number of trees. However, it was possible to achieve 100% accuracy with a peak list consisting of 608 peaks using the training set, which illustrates the problem of overfitting (Figure S4). To avoid overfitting, the regularization parameters must be added to the model, starting with the maximum depth (max-depth) parameter (Figure S5). The max_depth hyperparameter helps to regularize the model, and consequently, the model overfits less. Another important parameter is min_samples_leaf; it also serves as a regularization parameter (Figure S6). Overall, it was concluded that the optimal number of trees was n_estimators = 100, max_depth = 17, min_samples_leaf = 1, max_features = 20. In this case, the validation does not result in an accuracy gain, but we can significantly reduce overfitting while maintaining an accuracy above 94%.

The trends for validation curves have been illustrated above, but the optimal hyperparameters can also be found by cross-validated grid search over a parameter grid, cross-validated search over parameter settings (randomized search, Bayesian optimization, etc.), k-fold cross-validator, etc. Using the OOB estimation, which allows you to obtain an unbiased error estimate, it is theoretically possible to avoid using a separate test set or cross-validation to evaluate the model. However, OOB error estimation, while useful, is not always a substitute for full cross-validation, especially in the following cases: (1) the dataset has a complex structure or distribution that may not be fully accounted for by bootstrapping, (2) the evaluation of the model on a small dataset, where the OOB estimation may be unstable, and (3) it is necessary to carefully tune the hyperparameters of the model.

Due to the nature of the ToF-SIMS dataset, namely, the highly collinear data points (*m*/*z*), performing variable selection is beneficial before pattern recognition to obtain more robust and generalizable models. As mentioned above collinearity could potentially “dilute” the importance of features and complicate the interpretation of the results. When the feature number decreases, the feature importance, which was previously “diluted”, gets higher for the remaining ones (Figure S9). It can also be noticed that what can seem like a high-importance feature might be misleading. The collinearity between the fragments can also be decreased via a nested cross-validation process. Namely, an optimal number of variables can be determined using the CV to perform a cross-validated prediction performance of a model. This method assesses the prediction performance of models by sequentially reducing the number of predictors (ranked by variable importance) through a nested cross-validation process. In that case, the process is repeated N times using k-fold cross-validation (usually 5-fold), and in each step, the data was reduced, which resulted in an optimal number of variables. When collinear features are removed, the model accuracy should not be majorly affected. This also can be visualized as a boxplot graph of OOB and predictability accuracy of the RF dataset with all variables and with decreased numbers of variables.

It is possible to evaluate the importance of variables if the mean decrease in accuracy (MDA) was calculated in a permutation manner to determine each variable’s significance in the classification process. MDA considers the difference between the out-of-bag error resulting from randomly permuting variable values and the OOB from the original dataset. To obtain a more accurate understanding of the MDA for each variable, the variable selection for splitting decisions is usually repeated several times, and the average MDA was calculated. The variables with the highest MDA values can be retained in the final models, resulting in a reduced number of variables. Similarly, the reduced number of variables after the collinearity threshold was applied can be retained in the final model. The ion at *m*/*z* 885.62 has one of the highest predictor importance estimates of all the ions in the high mass range (Figure S11). Based on the previous publication, this is known to be a PI C18:0/C20:4 species that was identified as a potential marker for PAH.^144^ The classification accuracy for the entire dataset was assessed by constructing images where green pixels indicate healthy tissue and red pixels indicate unhealthy tissue (Figure 3). In the images, the control tissue primarily appears green, with a minority of pixels incorrectly classified as unhealthy. Conversely, in the PAH-affected arteries, red dominates, indicating the presence of the disease. The fact that some pixels are still misclassified highlights the importance of obtaining ground truth labels, which could enhance accuracy by refining the classification process and reducing misclassification.

## 4 Conclusion

The Random Forest algorithm proves to be a powerful tool for ToF-SIMS data, but there are some points of attention. Even though RF can handle outliers and noise in the data efficiently due to random sampling, the choice of descriptors for training is paramount in the analysis of ToF-SIMS data, highlighting the criticality of preprocessing. The algorithm is also prone to overfitting, especially on noisy data, necessitating validation. Moreover, as demonstrated, multicollinearity among features is an aspect to be addressed when working with ToF-SIMS data. If the datasets contain groups of correlated features that have similar significance for labels, then preference is given to small groups over large ones. Reducing multicollinear features can significantly improve the model’s performance, reliability, and interpretability by emphasizing the truly impactful variables and minimizing misleading feature importance. Identifying the most relevant features through nested cross-validation can help in selecting a subset of variables that enhances the model’s robustness. Finally, achieving relevant classification accuracy in ToF-SIMS analysis requires accurate ground truth labels. These labels can potentially enable a more precise evaluation and adjustment of the model.

On the other hand, it is resilient to common data challenges such as feature scaling and other monotonic transformations of feature values, due to the choice of random subspaces, which enhances its utility for ToF-SIMS datasets. RF is also worthwhile for its high parallelizability and scalability, making it suitable for the increasingly larger ToF-SIMS imaging datasets. The application of RF to ToF-SIMS imaging facilitates the classification of complex chemical compositions and the identification of significant features that contribute to these classifications. With consideration of the nature of the ToF-SIMS dataset, RF enhances the understanding of complex systems, paving the way for new applications in materials science, biology, and surface chemistry where detailed surface chemical mapping is essential.

## Supporting Information Available

Visualization of cross-validation technique and visualization of 5-fold cross-validation; visualization of leave-one-out cross-validation; examples of ToF-SIMS ion images; thresholded ion images; learning curves and model performance of Random Forest; figures of feature importance; table of samples used in the example (PDF)
