# Supplementary material for: The Application of a Random Forest Classifier to ToF-SIMS Imaging Data
Source: J Am Soc Mass Spectrom. 2024 Oct 25;35(12):2801–14. doi: 10.1021/jasms.4c00324 (PMC11622239; doi:10.1021/jasms.4c00324)
Supplement: Supplementary file 1 — js4c00324_si_001.pdf [file js4c00324_si_001.pdf]

Supporting Information

The Application of a Random Forest Classifier to ToF-SIMS Imaging Data

Mariya A. Shamraeva[1], Theodoros Visvikis[2], Stefanos Zoidis[2], Ian G. M. Anthony[1], Sebastiaan Van Nuffel[1, 2*]

[1]Maastricht MultiModal Molecular Imaging Institute (M4i), Maastricht University, Universiteitssingel 50, 6229 ER Maastricht, The Netherlands.

[2]Faculty of Science and Engineering, Maastricht University, Paul-Henri Spaaklaan 1, Maastricht 6229EN, The Netherlands.

*E-mail: s.vannuffel@maastrichtuniversity.nl

Table of Contents

Figure S1 2

Figure S2. 3

Figure S3 4

Figure S4 4

Figure S5 5

Figure S6 5

Figure S7. 5

Figure S8. 6

Figure S9. 6

Figure S10 7

Figure S11 7

Table S1 8

(a)

(b)

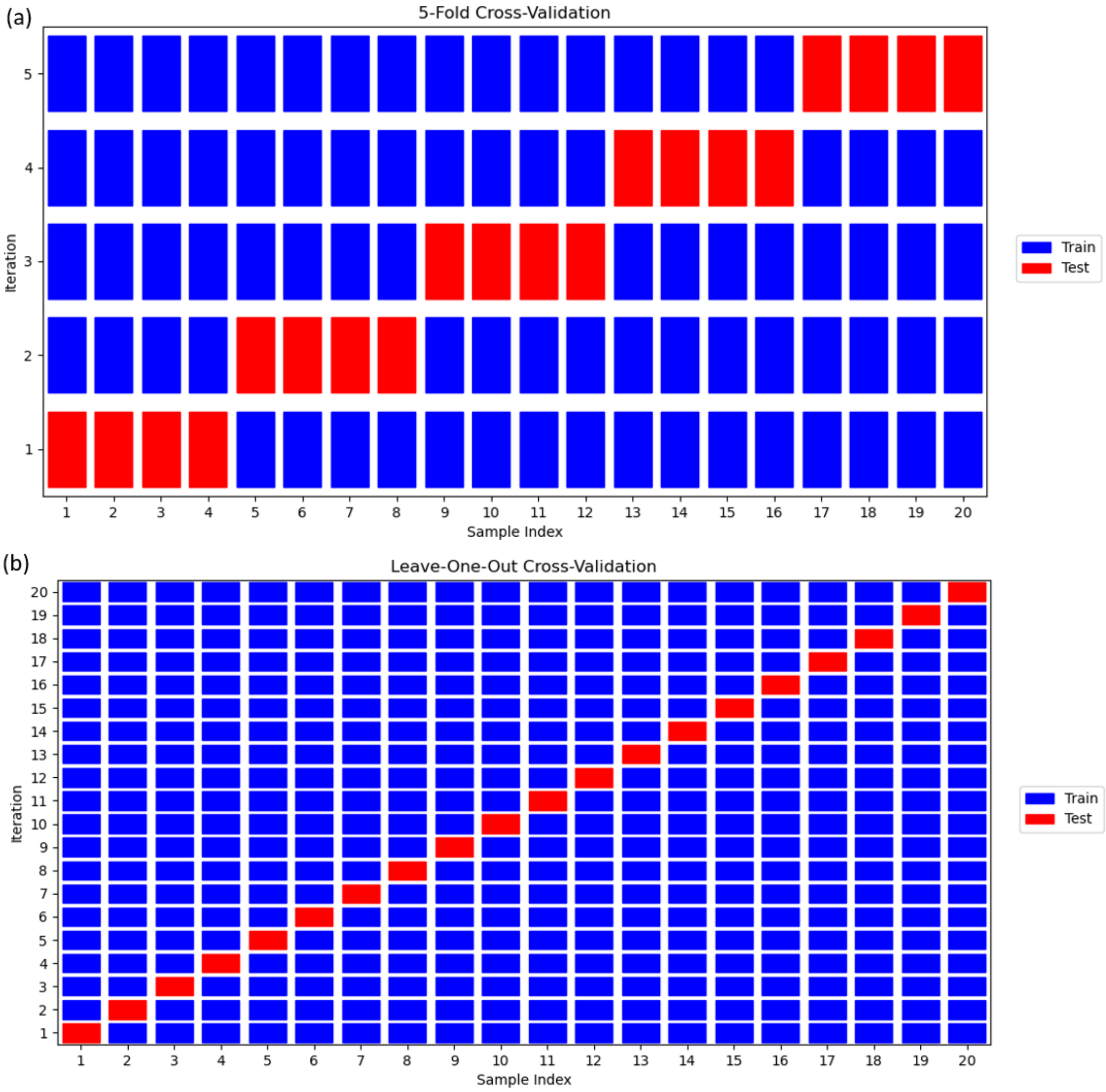

Figure S1. Cross-Validation Technique: In 5-fold cross-validation (a), the dataset is divided into 5 equal-sized subsets (folds). The model is trained and tested 5 times, each time using a different fold as the test set and the remaining 4 folds as the training set. The blue boxes represent the training data, and the red boxes represent the test data for each iteration. In leave-one-out cross-validation (b), the dataset is divided so that each data point serves as its own test set while the remaining data points form the training set. This process is repeated for each data point in the dataset, resulting in as many iterations as there are data points. Similar to 5-fold cross-validation, blue boxes denote the training data, and red boxes denote the test data for each iteration.

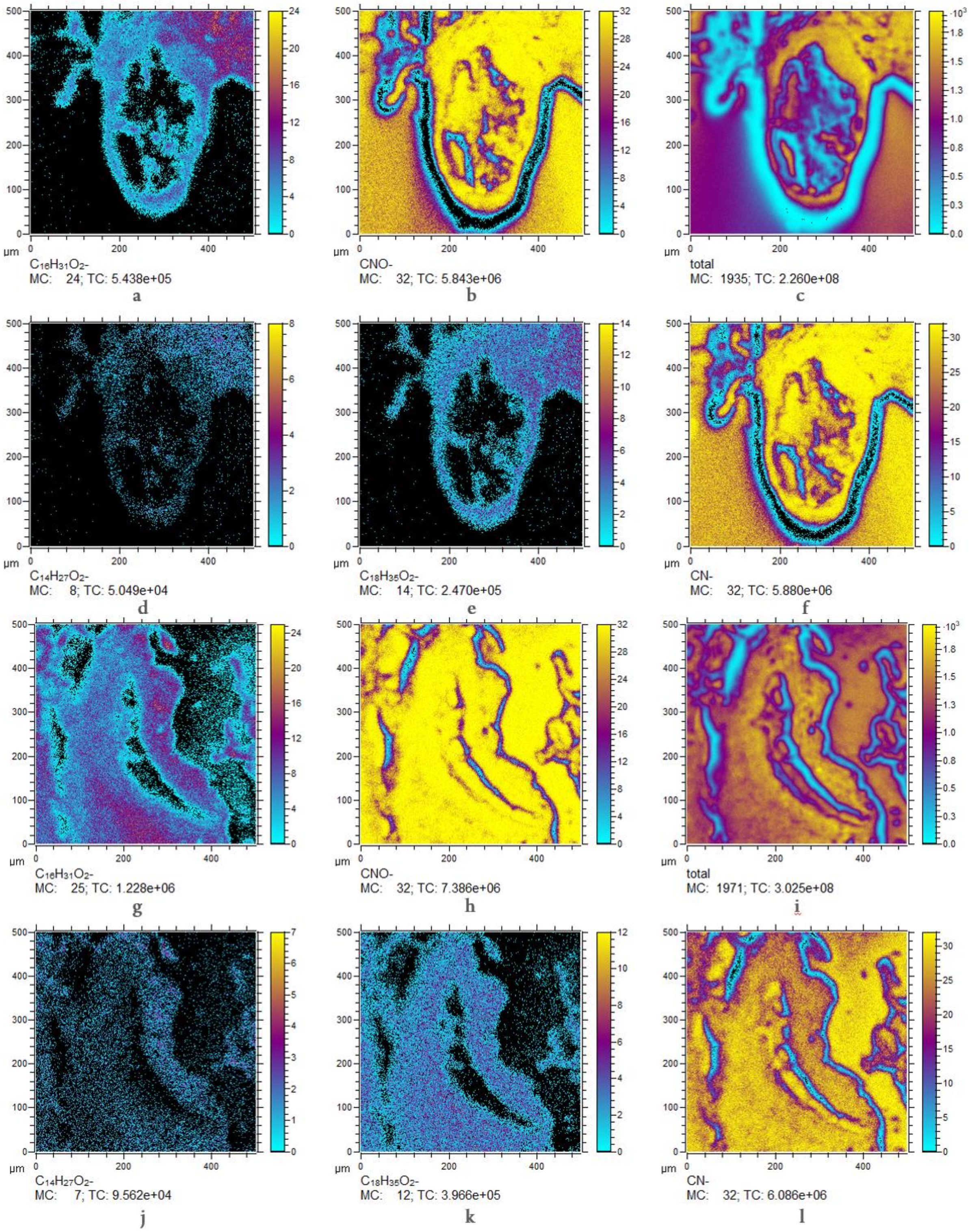

Figure S2. Example ToF-SIMS images of TIC and calibration ions CN^- (*m/z* 26.00), CNO^-(*m/z* 42.00), $C_{14}H_{27}O_2^-$ (*m/z* 227.20), $C_{16}H_{31}O_2^-$ (*m/z* 255.23), and $C_{18}H_{35}O_2$ (*m/z* 283.26) in negative ion mode for: a-f) pulmonary arterial hypertension arteries and g-l) control arteries.

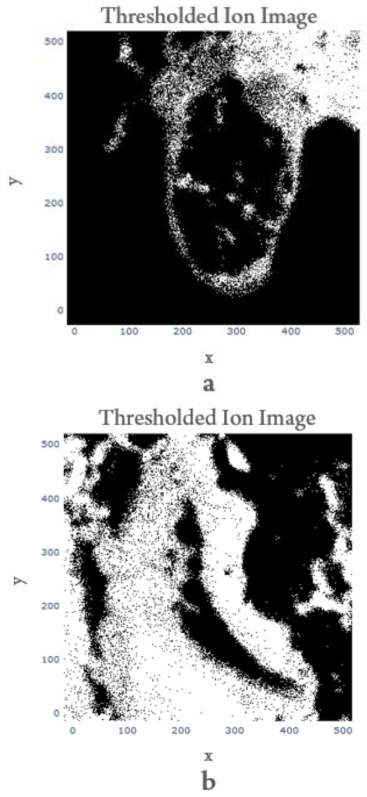

Figure S3. The ion images of palmitic acid ion (*m/z* 255.2), which was used as an indicator of biological tissue to remove background pixels from the image data with the tolerance of 0.5, and the threshold of 0.05 for a) pulmonary arterial hypertension arteries and b) control arteries.

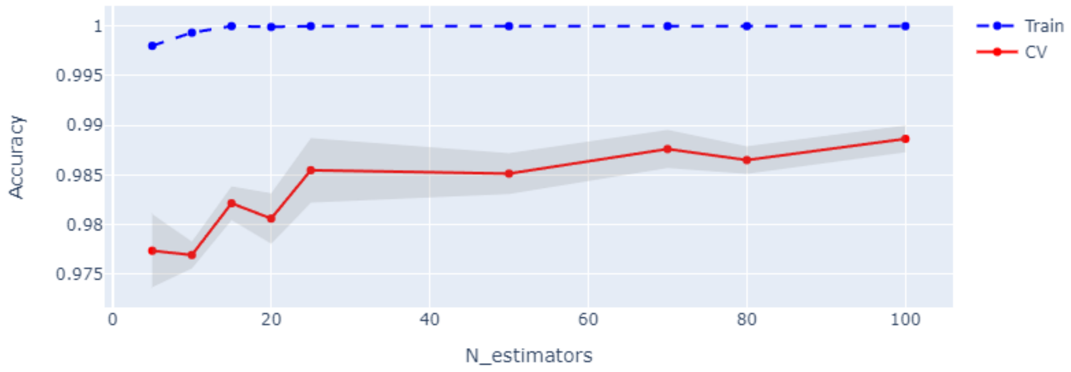

Figure S4. Learning curves of Random Forest performance for different n_estimators (the number of trees in the forest).

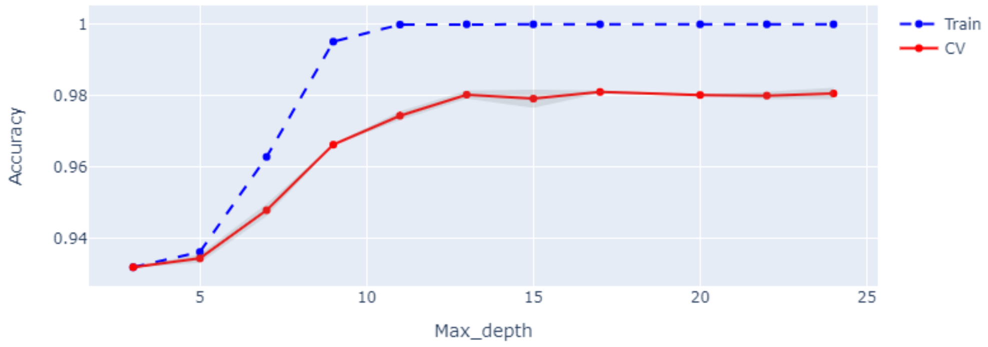

Figure S5. Learning curves of Random Forest performance for max_depth (the maximum depth of the tree).

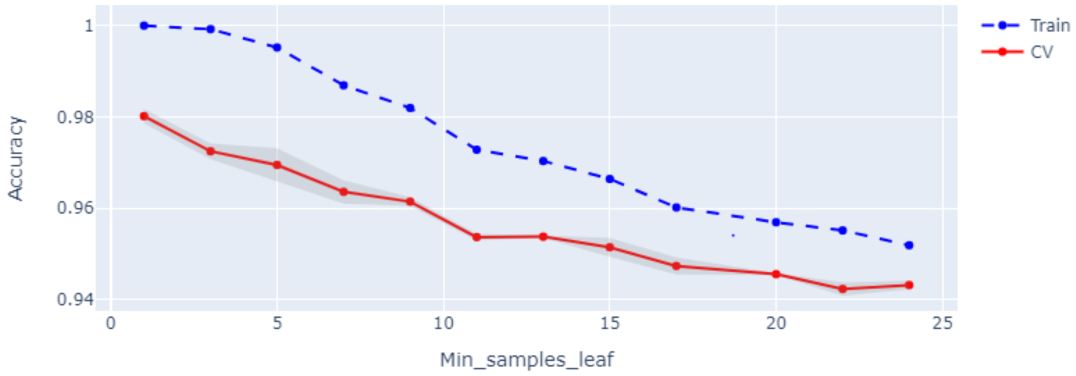

Figure S6. Learning curves of Random Forest performance for different min_samples_leaf (the minimum number of samples required to be at a leaf node).

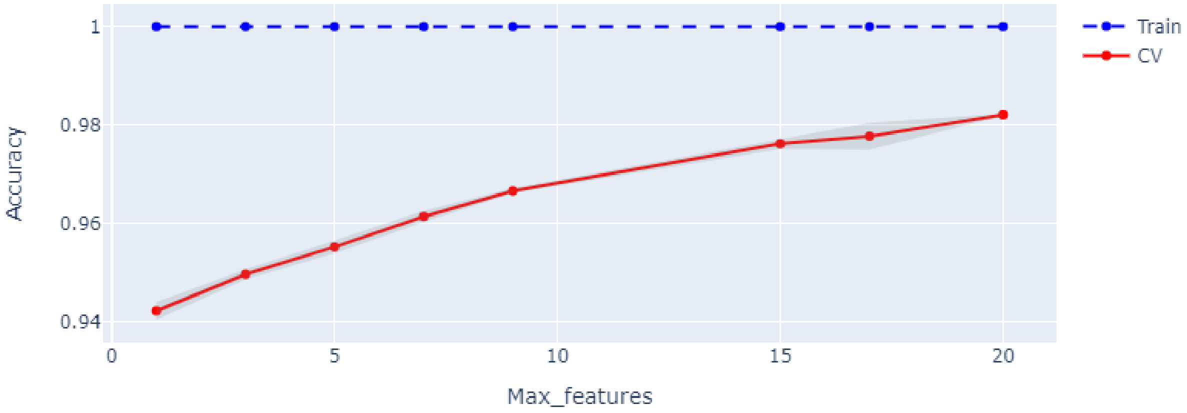

Figure S7. Learning curves of Random Forest performance for max_features (The number of features to consider when looking for the best split).

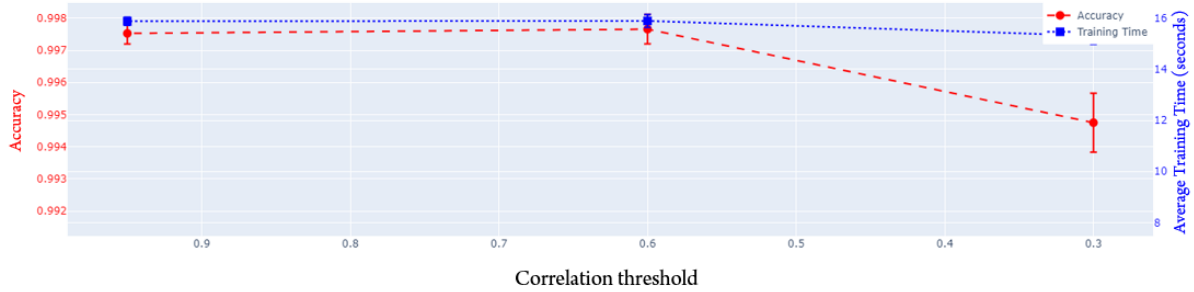

Figure S8. Model performance and training time across different collinearity thresholds.

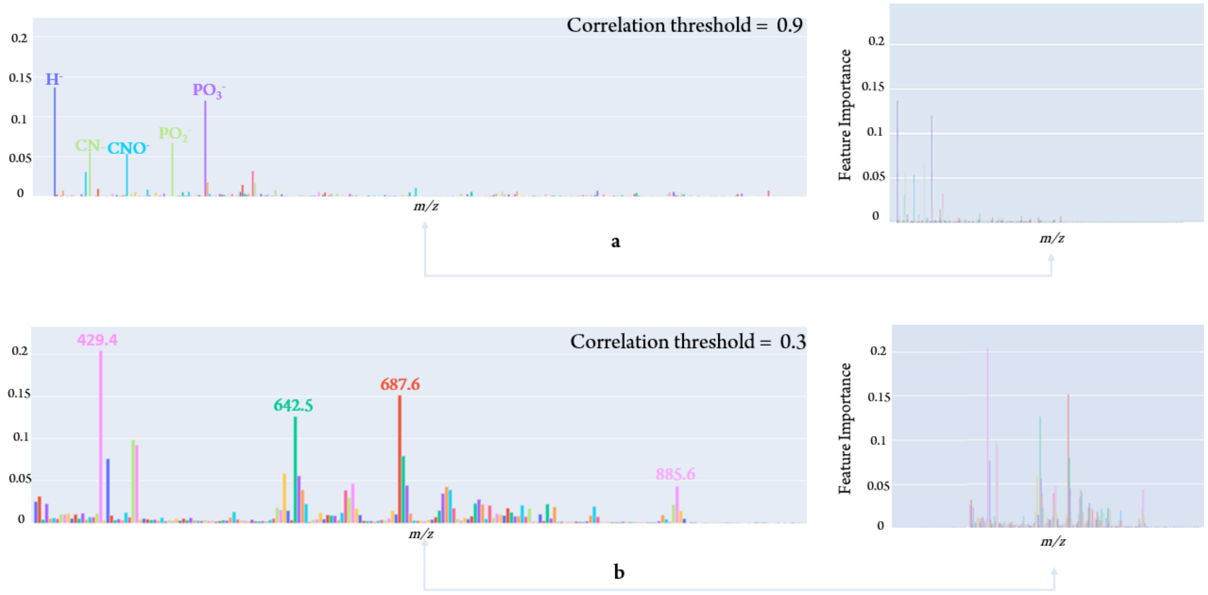

Figure S9. Change in feature importance across different collinearity thresholds: a) 0.9 and b) 0.3.

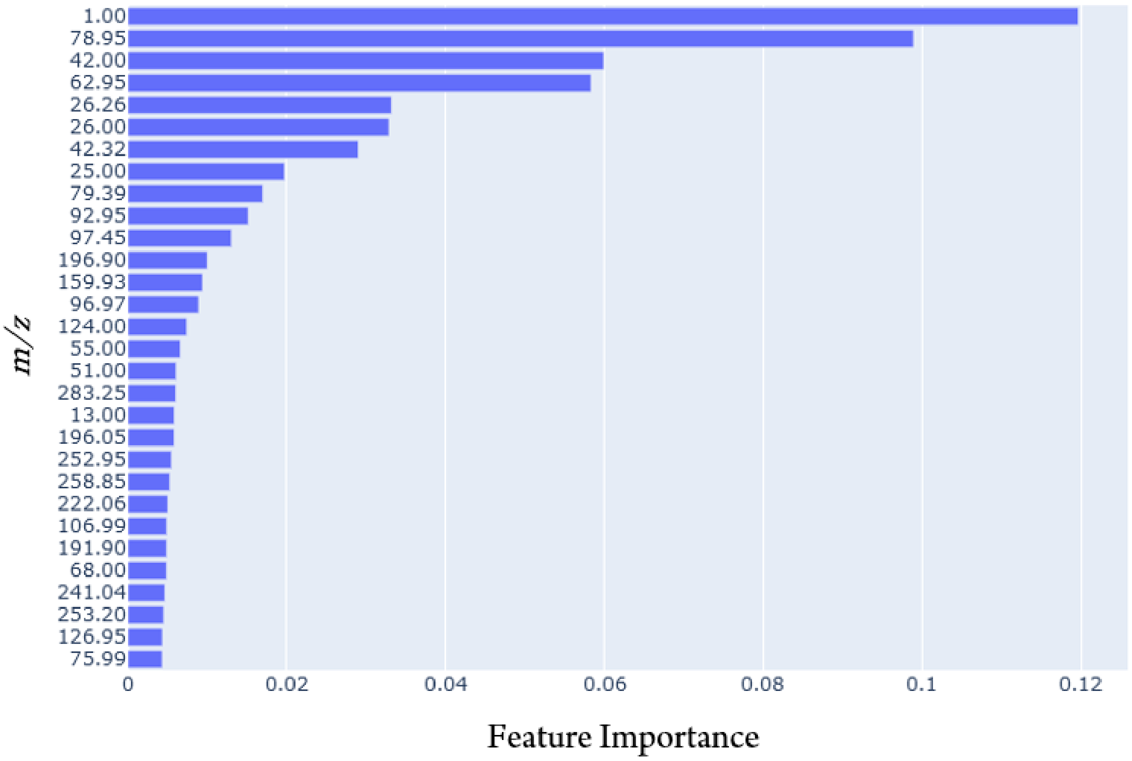

Figure S10. Top 30 important features before reducing the number of features.

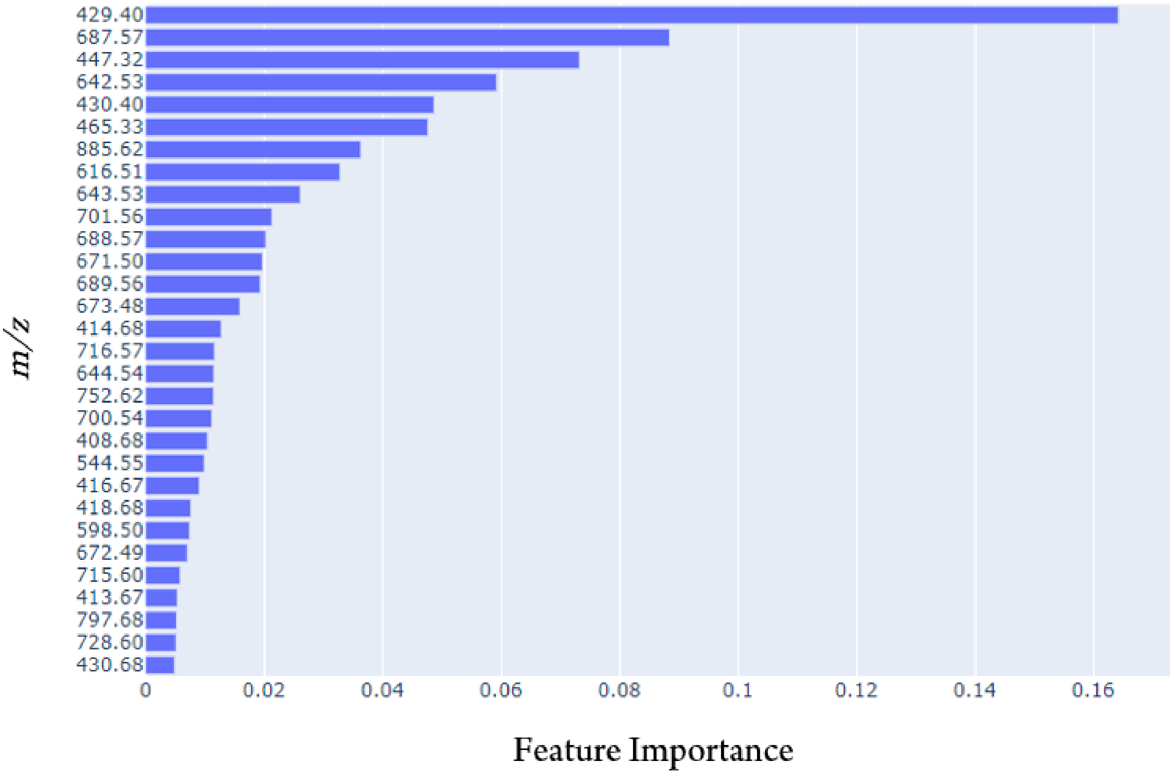

Figure S11. Top 30 important features after reducing the number of features.

Table S1. Lung samples of PAH samples and control samples.

	PAH/CTRL	(ext - int) / ext	Score	Bronchus
1	CTRL	0.310	1	N
2	CTRL	0.431	2	N
2	CTRL	0.431	2	N
4	CTRL	0.318	1	N
5	PAH	0.000	5	Bronchus
6	PAH	0.000	5	N
7	PAH	0.879	4	N
8	PAH	0.709	3	N
